# Impaired interactions of ataxin-3 with protein complexes reveals their specific structure and functions in SCA3 Ki150 model

**DOI:** 10.3389/fnmol.2023.1122308

**Published:** 2023-03-24

**Authors:** Piotr Piasecki, Kalina Wiatr, Milosz Ruszkowski, Łukasz Marczak, Yvon Trottier, Maciej Figiel

**Affiliations:** ^1^Institute of Bioorganic Chemistry, Polish Academy of Sciences, Poznań, Poland; ^2^Institute of Genetics and Molecular and Cellular Biology, University of Strasbourg, Illkirch, France

**Keywords:** SCA3, ataxin-3, CAG, polyQ, neurodegeneration, mouse mitochondria, aggregates, interactions

## Abstract

Spinocerebellar ataxia type 3 (SCA3/MJD) is a neurodegenerative disease caused by CAG expansion in mutant *ATXN3* gene. The resulting PolyQ tract in mutant ataxin-3 protein is toxic to neurons and currently no effective treatment exists. Function of both normal and mutant ataxin-3 is pleiotropic by their interactions and the influence on protein level. Our new preclinical Ki150 model with over 150 CAG/Q in ataxin-3 has robust aggregates indicating the presence of a process that enhances the interaction between proteins. Interactions in large complexes may resemble the real-life inclusion interactions and was never examined before for mutant and normal ataxin-3 and in homozygous mouse model with long polyQ tract. We fractionated ataxin-3-positive large complexes and independently we pulled-down ataxin-3 from brain lysates, and both were followed by proteomics. Among others, mutant ataxin-3 abnormally interacted with subunits of large complexes such as Cct5 and 6, Tcp1, and Camk2a and Camk2b. Surprisingly, the complexes exhibit circular molecular structure which may be linked to the process of aggregates formation where annular aggregates are intermediate stage to fibrils which may indicate novel ataxin-3 mode of interactions. The protein complexes were involved in transport of mitochondria in axons which was confirmed by altered motility of mitochondria along SCA3 Ki150 neurites.

## 1. Introduction

Spinocerebellar ataxia type 3, also known as Machado-Joseph disease (SCA3/MJD), is the most common type of dominantly inherited ataxias and belongs to a group of polyQ neurodegenerative disorders (McLoughlin et al., 2020). SCA3 is typically characterized by progressive motor incoordination and brain neuronal loss (Bettencourt and Lima, 2011; Koeppen, 2018) caused by the expansion of the CAG repeats in exon 10 of the *ATXN3* gene (>45) (Lima and Raposo, 2018). There is a substantial correlation between the number of CAG repeats, the age of onset of symptoms, and the severity of the disease course (Maciel et al., 1995; Leotti et al., 2021). The mutation results in an expanded polyQ tract in the ataxin-3 protein, which forms aggregates (Orr and Zoghbi, 2007). Ataxin-3 is a multifunctional protein and protease with a pleiotropic effect on many cellular pathways (Riess et al., 2008; Winborn et al., 2008; Grasty et al., 2019; Wiatr et al., 2019, 2021; Herzog et al., 2020). The pleiotropic influence results from interactions of both ataxin-3 and its aggregates with a plethora of other proteins, and these interactions may drive the disease. By interacting with K48-and non-canonical K63-linked ubiquitin chains, ataxin-3 plays diverse roles in ubiquitin signaling and protein quality control (Winborn et al., 2008; Todi et al., 2009, p. 3; Todi and Paulson, 2011; Carvalho et al., 2018). Furthermore, ataxin-3 regulates cytoskeleton function by interacting with dynein, an essential protein in retrograde axonal transport (Burnett and Pittman, 2005; Toulis et al., 2020). Ataxin-3 polyQ expansion results in either gain of function or loss of function of cellular processes and protein interactions (Paulson et al., 1997; Nascimento-Ferreira et al., 2011; Simões et al., 2012; Nóbrega et al., 2015). The pleiotropic function of the mutant protein (Wiatr et al., 2019, 2021) makes it difficult to untangle the molecular mechanisms of SCA3 and develop a treatment to prevent, cure, or stop the disease progression. Moreover, addressing the pathogenesis and the investigation of drug candidates requires a preclinical mouse model, which shows quick and intensive phenotype, and represents molecular, cellular, and motor changes mimicking SCA3. Phenotype acceleration in polyQ mouse models is always associated with a significantly greater number of CAGs in gene and glutamines in protein which results in an increasing number of aggregates.

We have previously shown a Ki91 model with 110–120 CAGs in the humanized *ATXN3* gene (Wiatr et al., 2019, 2021) with early inclusions and profound early proteome changes that play a crucial role in SCA3 pathogenesis. To demonstrate intensive SCA3 alterations and facilitate preclinical testing, we now generated a Ki150 knock-in mouse model containing 150–170 CAGs and obtained intensive SCA3-like symptoms. The Ki150 demonstrated dense and large aggregates, indicating intensive processes of protein aggregation and interactions. Protein interactions in SCA3 were previously investigated in isolated systems, in environments lacking large brain complexes or aggregates. To understand the SCA3 neuropathomechanism in our model, we determined the ataxin-3 interactions by identifying large complexes and proteins responsible for neuronal transport, mitochondria, and translation. We discovered that mutant ataxin-3 ultimately losses interaction with the complexes and that the marked feature of these complexes was their circular 3D structure. Guided by specific sets of interacting complexes and proteins, we assayed the physiological consequence in mouse neurons. We intensely investigated the functions of neurites in SCA3 by assesing live mitochondrial motility and mitochondrial transport in Ki150 neurites.

## 2. Results

### 2.1. Ki150 SCA3 mouse model contains mutant ataxin-3 with a large polyQ tract

**Figure 1:**
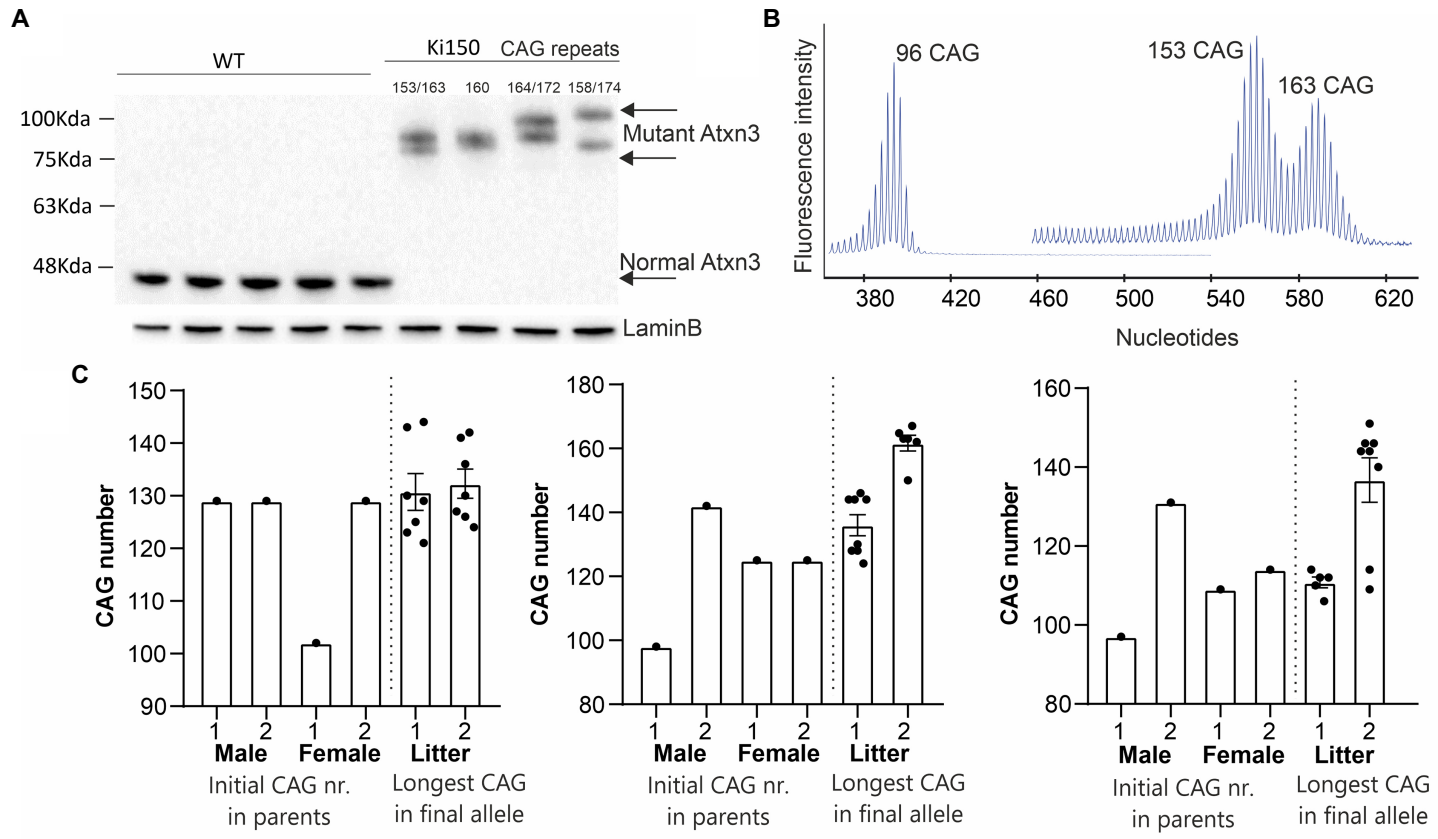
A new Ki150 mouse model expressing ataxin-3 with a large polyQ domain. Generation of the preclinical-type Ki150 mouse model with enhanced phenotype. **(A)** Homozygous Ki150 brain contains the polyQ-expanded ataxin-3 which reaches approximately 100 kDa in weight on immunoblot. The number of glutamines in ataxin-3 proteins is indicated in Ki150. LaminB was used as a loading control. **(B)** Representative examples of increasing CAG number in alleles of parents vs. offspring in humanized *ATXN3* gene between Ki91 and Ki150 mouse models. **(C)** Analysis of the CAG number (96 and 153–163 CAGs) in alleles visible on peeks representing the capillary electrophoresis of the DNA PCR products. Male and female denote parent animals containing the indicated CAG number in ATXN3 of parents’ allele. The “litter” denote the individual offspring mice with the CAG number of the final longest allele.

Expansion of the CAG repeats number in *ATXN3*, which occurs between generations (intergenerational expansion), usually correlates with the accelerated disease progression in SCA3 patients (Maciel et al., 1995; Leotti et al., 2021). Also, our Ki91 mouse showed intergenerational expansion (Switonski et al., 2015), resulting in a spontaneous increase in the number of CAG repeats in our mouse colonies, yielding mice containing between 110 and 130 CAG repeats. Therefore, our goal was to boost the phenotype further, to enhance the mouse model suitability for preclinical therapy testing. We used selective breeding of pairs with the highest number of CAGs repeats, reaching 150 units and above after multiple generations. Western blot analysis of the cerebral cortex of Ki150 mice showed differences in PAGE gel mobility of the ataxin-3 protein of various sizes (90–100 kDa) resulting from the number of glutamins between 153 and 174 (Figure 1A). Lamin B protein was used as a loading control. Figure 1B demonstrates capillary electroforesis detection of the DNA containing various numbers of CAGs in a PCR fragment resulting from Ki91 and Ki150 genome. The capillary electrophoresis demonstrates a dominant allele of 96 (Ki91), 153 and 163 (Ki150) and number of additional PCR products with lower or higher number of CAGs. Figure 1C demonstrates plots with examples of the CAG length of the longest and shortest allele in parents vs. offspring alleles (Figures 1B,C). The increase of variation in repeat numbers increase with generations, resulting in the identification of single expansions of 200 CAGs; however, the breeding of such animals was difficult.

### 2.2. The Ki150 mice produce robust inclusions across the brain

**Figure 2:**
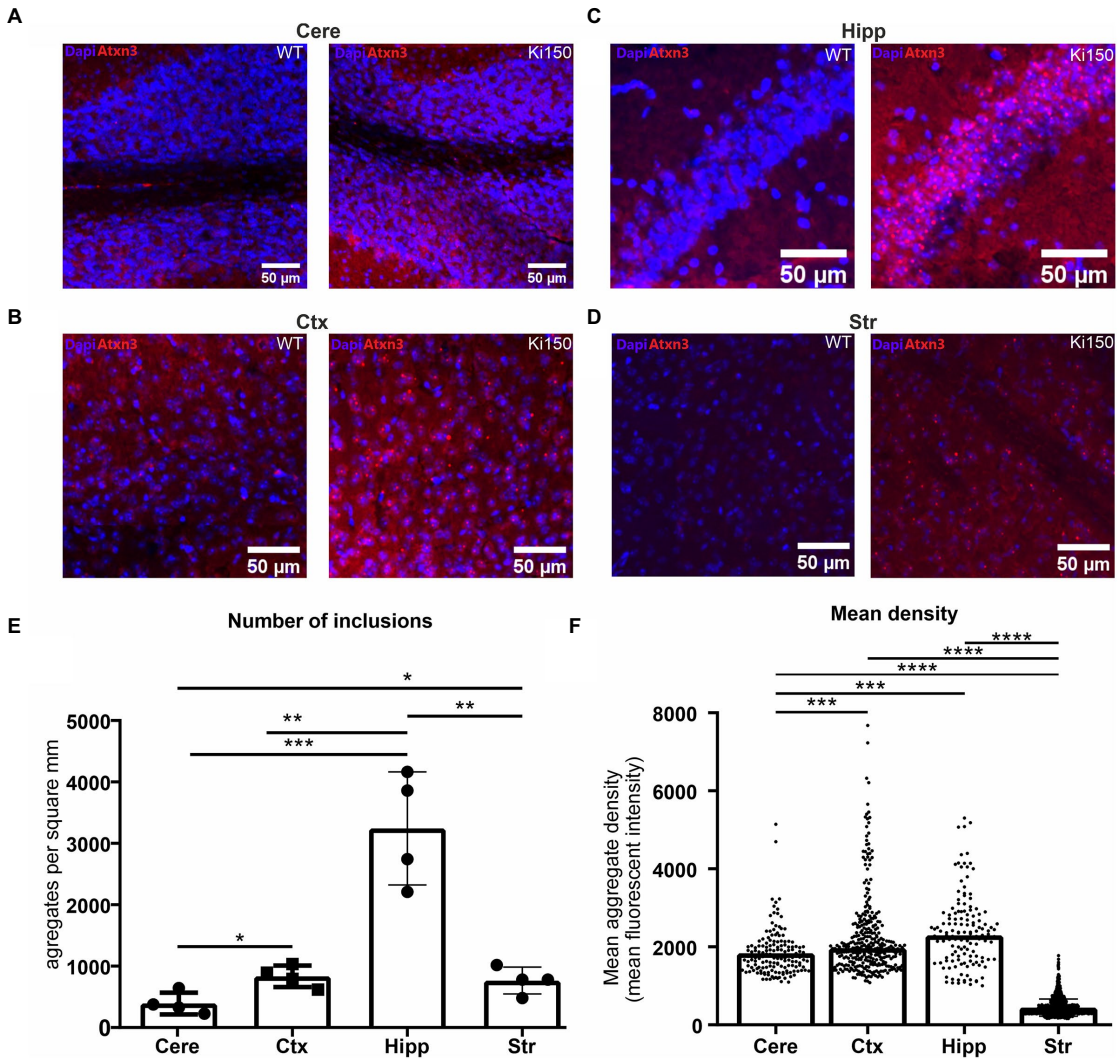
Ataxin-3-positive inclusions appear across the whole brain in SCA3 mouse model. **(A)** cerebellum (cere), **(B)** cerebral cortex (ctx), **(C)** hippocampus (hipp) **(D)** striatum (str). **(E)** Aggrecount imageJ macro identified about 400 inclusions per square millimeter in cerebellum, 800 inclusions per square millimeter in cerebral cortex, 3,000 inclusions per square millimeter in hippocampus and 760 inclusions per square millimeter in striatum (*n* = 4). **(F)** The density of a single inclusion was also the highest in the hippocampus (median = 2,298) as compared to cortex (median = 1969), cerebellum (median = 1727), and striatum (median = 0.382). Two-sample *t*-test (*p < 0.05, **p < 0.01, ***p < 0.001, ****p < 0.0001), error bars: SEM (*n* = 4 indicates 4 brains/4 mice. Per brain, 3 slices as technical replicates were used. Total number of 12 images were used for estimation of each brain region).

We have previously shown inclusions in the SCA3 Ki91 mouse occurring in all brain regions (Switonski et al., 2015; Wiatr et al., 2019, 2021). In the Ki150 mouse model, we observed the formation of larger inclusions in all brain regions and also in higher numbers than observed in Ki91 and at a younger Ki150 age. We quantified the number and density of aggregates in brain regions by Aggrecount Master macro (Klickstein et al., 2020) for Image J/Fiji (Figure 2). In Ki150 mice, we identified very large inclusions particularly apparent in brain regions with high cell density such as the hippocampus (Figure 2C). We also identified highest number of inclusions per mm^2^ (*n* = 4) in the hippocampus (mean = 3,243; SEM = 460.7) as compared to cortex (mean = 838.7; SEM = 87.6), cerebellum (mean = 393.1; SEM = 88.8), and striatum (mean = 766.1; SEM = 109.3) (Figure 2E). Additionally, we measured the fluorescent signal intensity of each inclusion from the sample (*n* = 4 and 3 replicates/slices per brain), which was equivalent to the median density of an inclusion (Figure 2F). The density of a single inclusion was also highest in the hippocampus (median = 2,298) as compared to the cerebral cortex (median = 1969), cerebellum (median = 1727) and striatum (median = 382.2). In summary, in the Ki150 mouse model, inclusions are visibly larger than in Ki91 and denser. In addition, a picture of entire slice of the Ki150 brain immunostained with anti-ataxin-3 antibodies demonstrate the overview of inclusion pathology throughout the entire brain and visible regions (Supplementary Figure 1A). Moreover, in Supplementary Figure 1B we included higher magnification images of ataxin-3 positive inclusion bodies in cerebellum, cerebral cortex, striatum, hippocampus, pons, and olfactory bulb.

### 2.3. Ki150 mice demonstrate a fast presentation and progression of a motor preclinical phenotype

**Figure 3:**
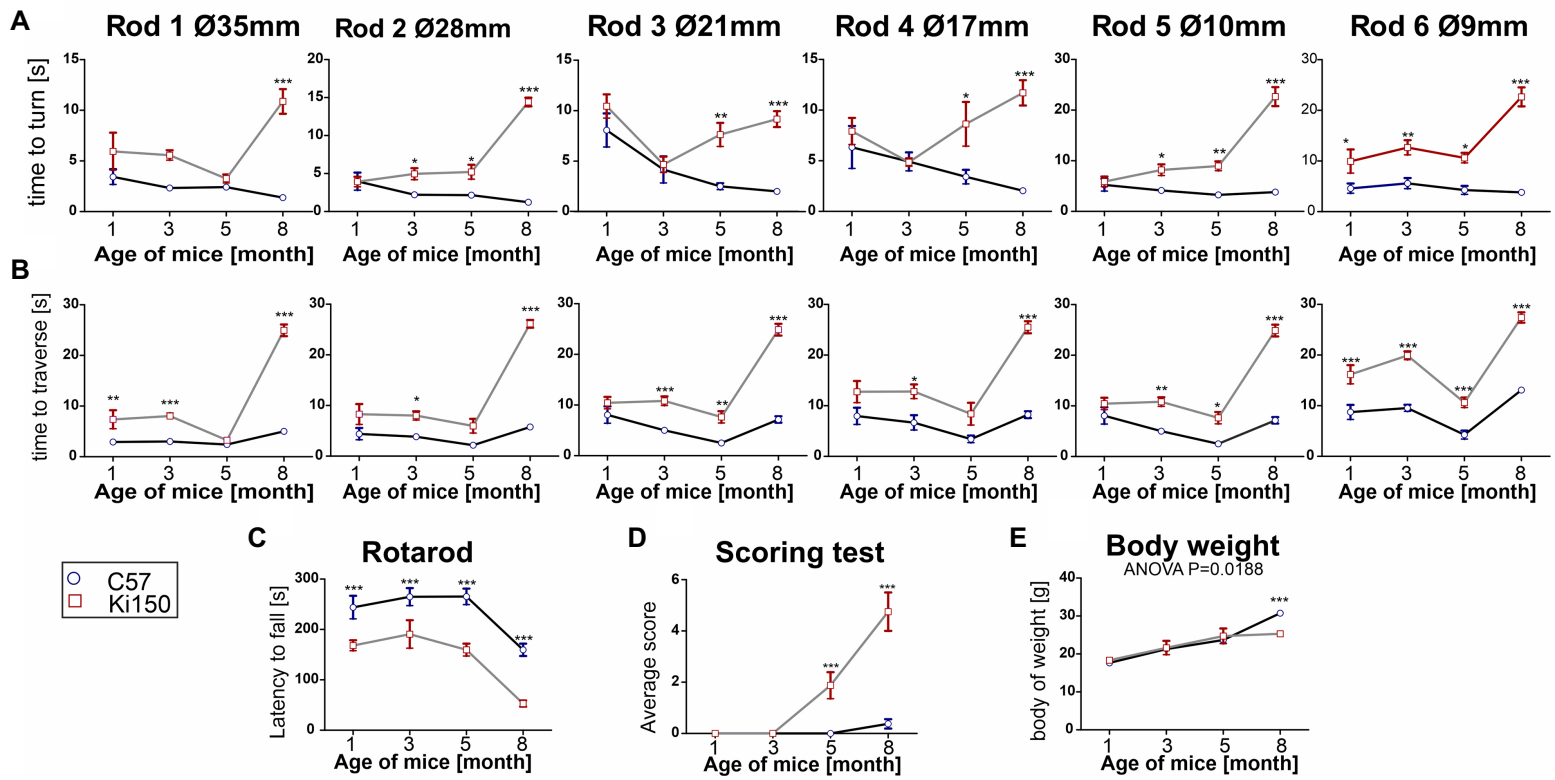
A progressive motor decline in Ki50 SCA3 knock-in model presents fast and severe phenotype. In the elevated beam walk test **(A,B)** “time to turn” and “traverse time” were measured on six rods with decreasing diameter (diameter of rods are indicated by Ø in mm). **(A)** One-month-old animals needed more time to turn on the rod 6 representing the highest level of difficulty and with disease progression more rods presented a challenge to Ki150 mice; Ultimately, at the age of 8 months Ki150 mice needed more time to traverse on all rods. **(B)** Similarly to the “time to turn” 1-month-old Ki91 mice needed significantly more time to traverse on rod 6, but also 1, whereas older 8-month-old mice needed more time on all rods. **(C)** Motor incoordination in accelerated rotarod (4–40 rpm in 5 min) was presented by Ki150 already at the age of 1 month. **(D)** In the scoring test, 5-month-old Ki150 mice presented SCA3 phenotype: incoordination, gait disturbances, kyphosis, and hind limb clasping. **(E)** The reduction of body weight gain in Ki150 was observed at the age of 8-month. Two-way ANOVA for every test besides body weight was *p* < 0.001. Two-way ANOVA with Bonferroni *post hoc* test (*p* ≤ 0.05; total number of biological replicates: *n* = 16, *n* = 8 per genotype), error bars: SEM. Asterisks denotes a two-way ANOVA (*p < 0.05, **p < 0.01, ***p < 0.001).

In order to determine behavioral phenotypes characteristic of SCA3 in Ki150 mice, we performed several tests measuring motor performance. We discovered that first motor symptoms occur already in 1-month-old Ki150 animals (*n* = 8 per genotype). Already at this young age, Ki150 mice performed worse than WT in the elevated beam walk and Rotarod test, presenting loss of balance and incoordination (Figures 3A–C). Ki150 mice needed more time to turn on a rod (diameter: 9 mm) (Figure 3A) and took more time to traverse rods (diameter: 35 and 9 mm) in the elevated beam walk test (*p* < 0.05; two-way ANOVA, Bonferroni) (Figure 3B). With the progression of SCA3, the time needed for turning on a rod and traverse was significantly longer ultimately on all tested rods (diameter: 35, 28, 21, 17, 10, and 9 mm) (*p* < 0.05; two-way ANOVA, Bonferroni; Figures 3A,B). Moreover, Ki150 commit more foot slips while traversing rods. In addition, the incoordination observed in the Rotarod was observed starting from the age of 1-month and progressed with age (*p* < 0.001; two-way ANOVA, Bonferroni; Figure 3C). 5-month-old Ki150 mice showed deterioration of motor phenotype in the scoring test, which evaluated gait, motor coordination, balance, hindlimb clasping, and kyphosis (*p* < 0.0001; two-way ANOVA, Bonferroni) (Figure 3D). At the age of 8-months, Ki150 also demonstrated reduced body weight (*p* < 0.0001; two-way ANOVA, Bonferroni) (Figure 3E). There was no correlation between body weight and behavioral tests at any age (*p* < 0.05, correlation test).

### 2.4. Ki150 brain reveals the presence of mutant ataxin-3 in fractions containing subunits of large protein complexes

We have previously demonstrated that humanized mutant ataxin-3 from Ki91 model influenced very broad changes in protein levels and their phosphorylation. Since the protein from Ki150 contains an even more expanded polyQ tract and produces very dense brain inclusions, we decided to characterize the protein and the complexes to a greater extent. We asked how the protein interacts with other proteins and how large and stable are the complexes interacting with ataxin-3 containing a large polyQ domain. To separately analyze the influence of mutant or normal ataxin-3 on the complex formation, we took advantage of the fact that our models contain homozygous alleles (Ki150/150 or Ki21/Ki21).

**Figure 4:**
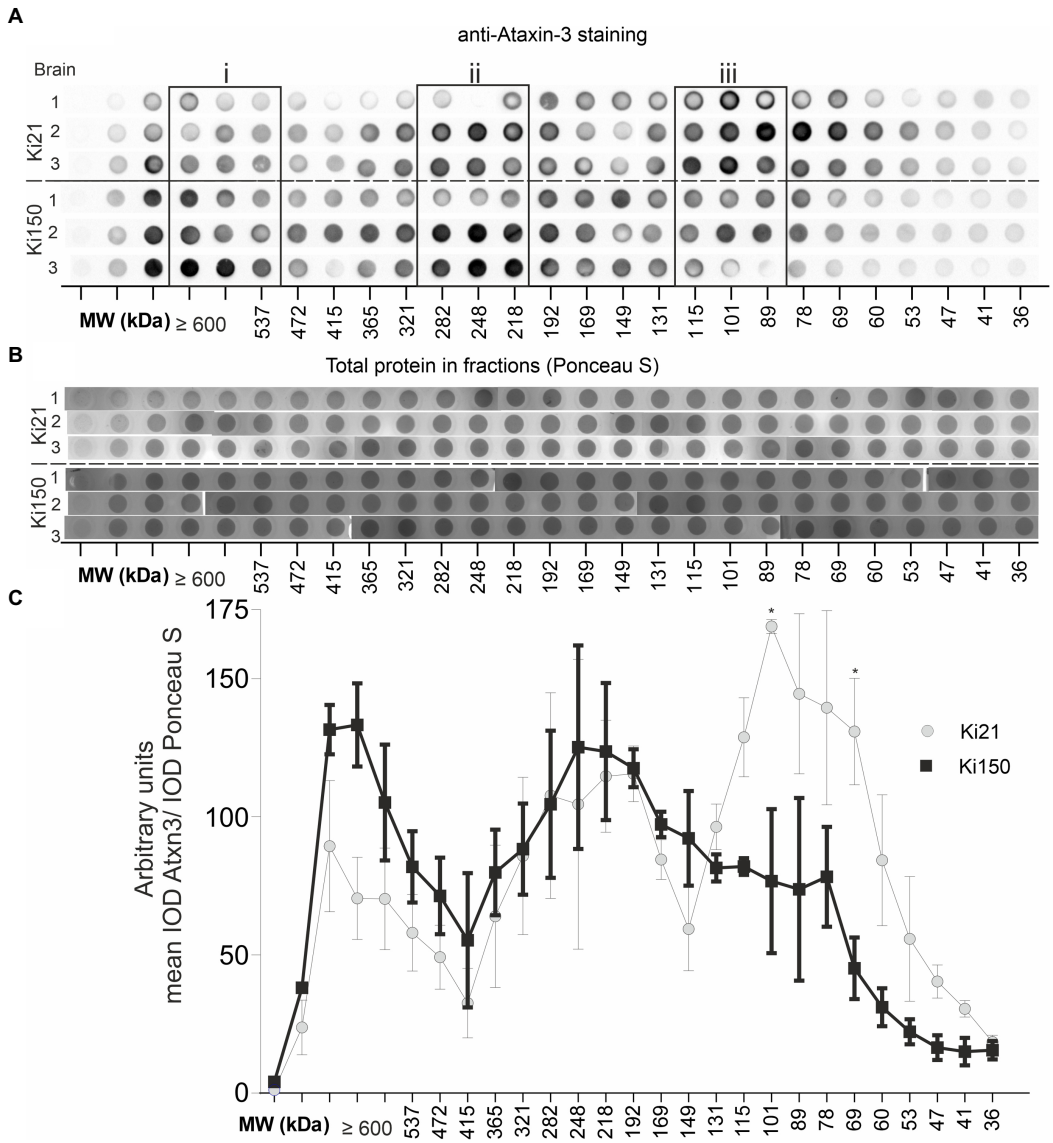
Ataxin-3 is distributied in large protein complexes after chromatographic fractionation. **(A)** Whole brain lysates after fractionation using size exclusion chromatography (SEC). Protein complexes were separated on the basis of their size (in the range of 600–22 kDa) and divided into 31 fractions. Dot blots were probed with an anti-ataxin-3 antibody. The signal indicates the distribution of ataxin-3 among the fractions of the protein complexes in Ki21 and Ki150 protein complexes. **(B)** Ponceau-stained vacuum dot blot of protein fractions corresponding to the size range >600–22 kDa. **(C)** Based on the dot blot results, we can distinguish 3 peaks; the first (>600 kDa), the second (248–192 kDa), and the third (115-89 kDa). In the third peak (115–89 kDa) the level of mutant ataxin-3 in Ki150 brains is significantly lower than in the control Ki21 brain lysates. Two-sample *t*-test (*p < 0.05, **p < 0.01, ***p < 0.001), error bars: SEM total number of biological replicates: *n* = 6, *n* = 3 per genotype.

Therapeutic protein characterization often involves two or more or more orthagonal chromatographic methods. In our setup, we used orthogonal chromatographic methods in native conditions to resolve the protein brain lysates from Ki150 containing human mutant ataxin-3 with around 150Q and Ki21 containing human normal ataxin-3 21Q. First, we separated the proteins by charge using ion exchange chromatography (IEC) and then by size using size exclusion chromatography (SEC). We aimed to (i) investigate the distribution of ataxin-3 protein in SEC fractions, (ii) identify coeluting ataxin-3 partners, and (iii) assess the physical properties of putative mutant and normal ataxin-3 complexes In IEC, ataxin-3 was present in the flowthrough, likely due to the relatively high ionic strength of the PBS buffer (Supplementary Figure 2A). Nonetheless, IEC removed fat and nucleic acids, which was a necessary step prior to SEC. The SEC fractions contained native soluble proteins and preserved their interactions thanks to using PBS-EDTA buffer for SEC. The total protein concentration in the selected fraction (A280) was different for both genotypes. The protein concentration in ≥600-kDa fractions was higher in Ki150 samples, while protein concentration in 115–89 kDa fractions was higher in Ki21 samples (Supplementary Figure 1B). A vacuum dot-blot was performed and immunostained with anti-ataxin-3 antibodies to determine the ataxin-3 distribution in protein fractions from Ki150 and Ki21 brains (Figure 4A). The integrated optical density (IOD) of the ataxin-3 signal from dot blots was plotted against the SEC-calculated molecular weight within fractions. Additionally, after vacuum dot-blot Ponceau S staining was performed for loading control (Figure 4B). The plot demonstrated three peaks of the highest signal indicating a prominent presence of ataxin-3 in (i) ≥600 kDa, (ii) 282–218 kDa protein fractions in both genotypes, and markedly higher level of normal ataxin-3 in (iii) 115–89 kDa fraction (Figure 4C). The predicted sizes of fractions are not equivalent to the molecular weight for monomeric normal (48 kDa for Ki21) and mutant ataxin-3 (95 kDa for Ki150), suggesting that ataxin-3 forms extensive complexes with other proteins or forms homooligomers.

### 2.5. Fractions with mutant ataxin-3 show depletion or enrichment of proteins involved in large molecular complexes regulating aggregation, transport and CamK proteins

**Figure 5:**
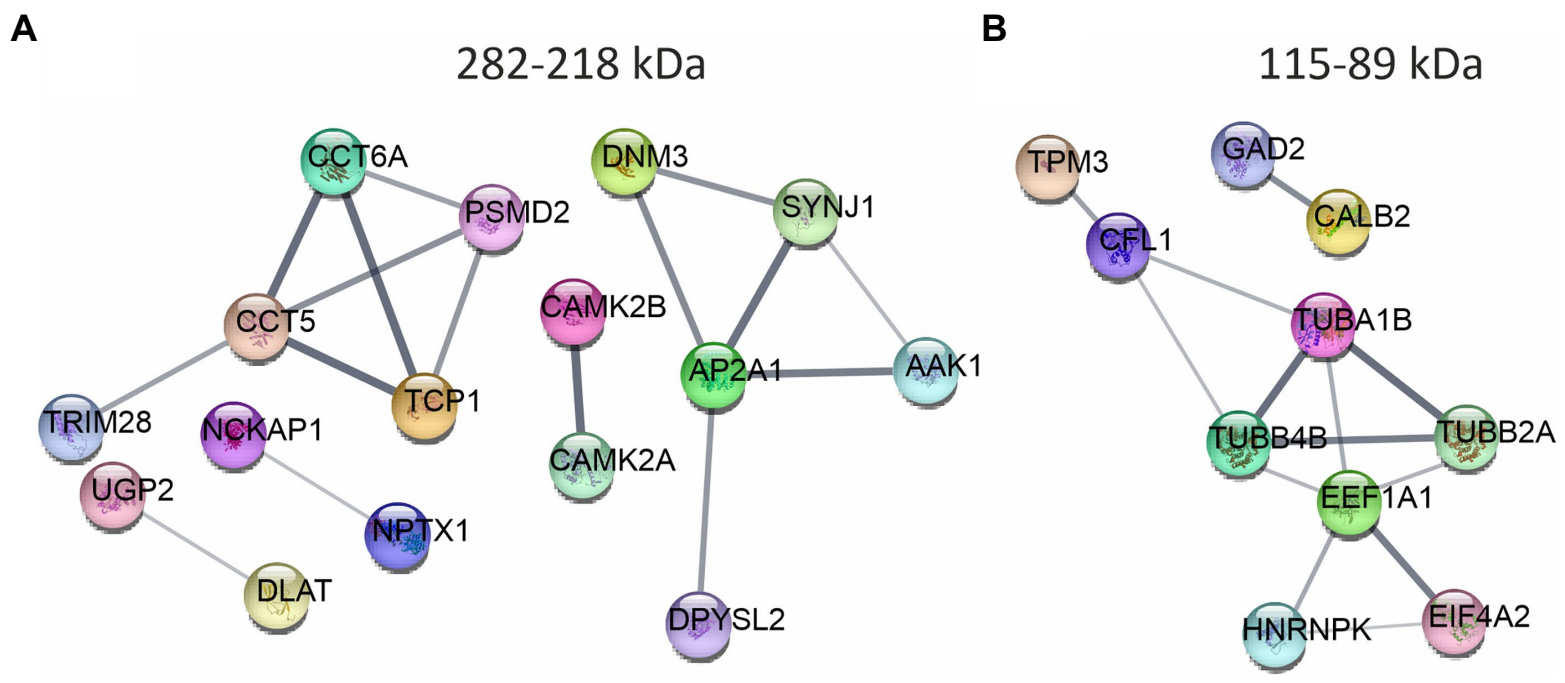
The networks of proteins composing complexes with ataxin-3 identified by ion exchange chromatography (IEC) and size using size exclusion chromatography (SEC) followed by LC–MS/MS. The networks were generated using String database and clustering (STRING Network *p* < 10e-16) ( https://string-db.org/ ). **(A)** The fraction 282–218 kDa contains highly downregulated Camk2a, CamK2b, proteins belonging to Chaperon Containing TCP1-complex, and proteins being part of the adaptor protein 2 complexes. **(B)** In the fraction 115–89 kDa proteins responsible for the translation initiation process: Hnrnpk and Eif4a2 were highly enriched in the Ki150 brains. Proteins characteristic for the GABAergic neurons Gad2, Calb2 and Pcp2 were also altered. *N* = 3.

We traced the interaction of proteome in the presence of mutant or normal ataxin-3 to reveal the potential partners in the brain of homozygous Ki21 and Ki150. The collected fractions (ii) 282–192 kDa, and (iii) 115–89 kDa positive for ataxin-3 protein (by dot blot) were subjected to protein identification and quantitation by MS. The fraction (i) ≥600 kDa turned out to be unsuitable for MS analysis since it may contain aggregates inaccessible for trypsin and high content of peptide fragments that constitute background on MS spectrum. However, by comparing Ki21 and Ki150 samples from fractions (ii) and (iii), we identified the depletion or enrichment of proteins (*p* < 0.05; two-sample *t*-test; Figure 5) that are part of specific cellular families of proteins and complexes. In fractions 282–218 kDa we found that proteins CCT5 and 6, and Tcp1 involved in Chaperon Containing TCP1-complex (CCT complex; T-complex), responsible for the regulation of protein folding, aggregation, and transport along neurites, were highly depleted in Ki150 brain fractions vs. Ki21 fractions (Table 1; Figure 5A). Also, the Ap2a1 and Aak1 (AP2-associated protein kinase 1), which are part of adaptor protein 2 complexes and together with Synj1 are responsible for vesicular transport, were depleted in Ki150 fractions (Table 1; Figure 5A). The most depleted proteins in Ki150 brain fractions were Camk2a and Camk2b kinases responsible for calcium homeostasis (Table 1; Figure 5A). The proteins highly enriched in 115–89 kDa Ki150 brain fractions were Hnrnpk and Eif4a2 involved in the translation initiation process (Table 2; Figure 5B). We also identified highly depleted Gad2, the GABA synthesizing enzyme, Calb2, a calcium-binding protein, and PCP2 proteins (Table 2; Figure 5B), all characteristic of GABAergic neurons such as Purkinje neurons in the cerebellum.

**Table 1:** Proteins depleted in Ki150 fractions 282–218 kDa positive for ataxin-3 protein compared to Ki21 control.

Proteins by gene acronim fraction: 282–218 kDa	Name	Student’s *T*-test *p*-value	Student’s *T*-test difference
Camk2b	Calcium/calmodulin-dependent protein kinase type II subunit beta	7.56E-05	−18.724
Camk2a	Calcium/calmodulin-dependent protein kinase type II subunit alpha	0.00030	−18.082
Nckap1	Nck-associated protein 1	0.00605	−11.789
Cct6a	T-complex protein 1 subunit zeta	0.00605	−12.009
Aak1	AP2-associated protein kinase 1	0.01448	−12.912
Nptx1	Neuronal pentraxin-1	0.02229	−9.112
Ap2a1	AP-2 complex subunit alpha-1	0.02229	−9.295
Psmd2	26S proteasome non-ATPase regulatory subunit 2	0.02230	−9.293
Dlat	Dihydrolipoyllysine-residue acetyltransferase component of pyruvate dehydrogenase complex. Mitochondrial	0.02231	−9.755
Tcp1	T-complex protein 1 subunit alpha	0.02233	−9.830
Synj1	Synaptojanin-1	0.02540	−10.568
Ugp2	UTP–glucose-1-phosphate uridylyltransferase	0.04267	−10.681
Cct5	T-complex protein 1 subunit epsilon	0.04395	−9.894
Dnm3	Dynamin-3	0.04742	−10.011
Trim28	Transcription intermediary factor 1-beta	0.04984	−9.135

**Table 2:** Proteins with altered levels in Ki150 fractions 115–89 kDa positive for ataxin-3 protein compared to Ki21 control.

Proteins by gene acronim fraction: 115–89 kDa	Gene name	Student’s *T*-test *p*-value	Student’s *T*-test difference
Hnrnpk	Heterogeneous nuclear ribonucleoprotein K	0.03204	9.168
Aldh6a1	Methylmalonate-semialdehyde dehydrogenase [acylating]. mitochondrial	0.0332	−8.259
Gad2	Glutamate decarboxylase 2	0.03325	−7.953
Tpm3	Tropomyosin 3	0.03333	8.944
Pcp2	Purkinje cell protein 2	0.03588	10.322
Calb2	Calbindin 2. isoform CRA_a	0.04949	6.770
Eif4a2	Eukaryotic initiation factor 4A-II	0.04951	6.907
Hp	Haptoglobin	0.04982	7.772

### 2.6. Mutant ataxin-3 interactors form sets of proteins that are functionally associated with mitochondria and translation

Molecular complexes identified in fractions 282–218 kDa such as CCT complex, AP2 and the family of Ca++ kinases usually bind a variety of other cellular proteins that tune their physiological function. Therefore, we used a co-immunoprecipitation assay with specific anti-ataxin-3 antibody on magnetic beads to identify the interacting proteins. We aimed to cross-correlate our findings of large molecular complexes and to further characterize the physiological and pathogenic mechanisms behind complex interactions of mutant and normal ataxin-3 in brain tissue. The immunoprecipitation and subsequent proteomic identification and quantitation involved brains from Ki21 knock-in mouse model, and the Ki150 knock-in previously used for SEC and IEC experiment. Artefactual interactors that may bind to dynabeads or antibodies were identified and excluded from the analysis by using immunoprecipitation on brain lysates with dynabeads coupled with mouse control IgG (Supplementary Tables 1, 2).

**Figure 6:**
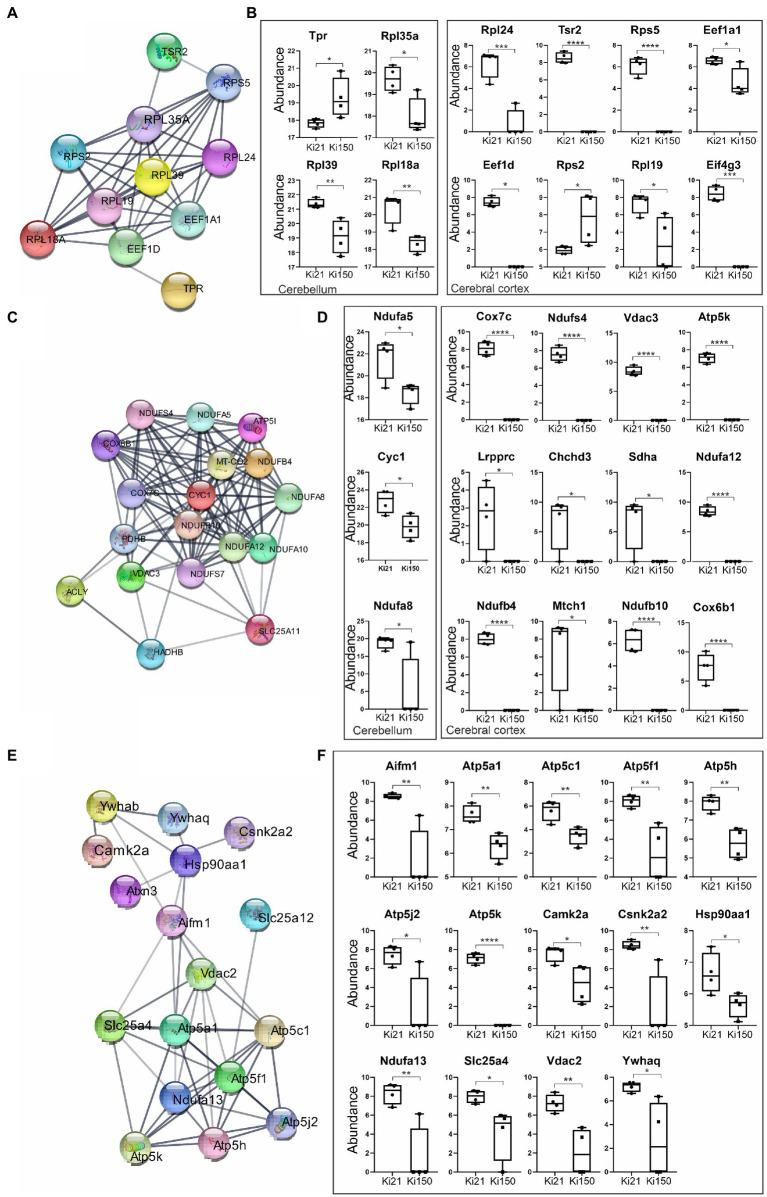
IP-LC–MS/MS proteomic analysis of ataxin-3 partners reveals 3 sets of proteins with significantly weakened interaction with the mutant ataxin-3 in the Ki150 SCA3/MJD cerebellum and cortex. The network of proteins displaying weakened interaction with the ataxin-3 containing expanded polyQ tract in the cerebellum **(A,C)** and cerebral cortex **(A,C,E)** of Ki50 mice were generated using String database and clustering (STRING Network *p* < 10e-16) (https://string-db.org/). Diagrams demonstrate relative levels of proteins after their pull-down and proteomic analysis (Ki150 vs. Ki21) reflecting the change or even complete loss of protein interaction with mutant vs. normal ataxin-3 in the cerebellum and cerebral cortex (**B,D,F**; *p* < 0.05; two-sample *t*-test). **(A,B)** The first cluster contained proteins related to the translation in the cerebellum and cerebral cortex. **(C,D)** The mutant ataxin-3 demonstrated very weak interactions with mitochondrial proteins (second cluster) in the Ki150 cerebellum and Ki150 cerebral cortex. **(E,F)** The third set of proteins with diminished interaction with mutant ataxin-3 (150 CAG repeats) in the cerebral cortex is involved in mitochondrial transport. Two-sample *t*-test (*p* ≤ 0.05; *p < 0.05, **p < 0.01, ***p < 0.001); total number of biological replicates: *n* = 8, *n* = 4 per genotype; error bars: SEM.

We identified 394 and 47 ataxin-3 interactors in cerebral cortex and cerebellum, respectively, that were significantly dysregulated in 2-month-old Ki150 vs. Ki21 (*p* < 0.05; two-sample *t*-test; Supplementary Tables 3, 4). The network of functionally interconnected protein interactors of ataxin-3 was demonstrated by STRING (Figures 6A,C,E; PPI *p* < 10e-16). The diagrams demonstrate relative levels of proteins after their pull-down and subsequent quantification by proteomics in Ki150 vs. Ki21, reflecting the change in protein–protein interactions between mutant vs. normal ataxin-3 (Figures 6B,D,F; *p* < 0.05; two-sample *t*-test). The identified proteins belong to 3 functional clusters, and they showed considerably weakened or even lack of interaction with mutant ataxin-3 in Ki150 vs. Ki21 after pull-down. The first protein cluster contained proteins related to translation both in cerebellum (Rbm8a, Rpl39, Eef1a1, Rpl18a, Tpr, Rpl35a, Rps2, Hnrnpd, Rpl19, Eif5a;) and cortex (Eef1a2, Eef1d, Eif4g3, Tsr2, Rpl24, Rps5) (quantification: proteins pull-down and proteomics, *p* < 0.05; two-sample *t*-test; STRING Network *p* < 10e-16; Figures 6A,B). Another two functional clusters were related to mitochondria and their transport in neurons. The mutant ataxin-3 from Ki150 demonstrated very week interactions vs. normal ataxin-3 from Ki21 with mitochondrial proteins in the cerebellum (Cyc1, Ndufa5, and Ndufa8, Figure 6C) and the cerebral cortex (Atp5k, Acly, Cox7c, Cox6b1, mt-Co2, Ndufa10, Ndufa12, Ndufb10, Ndufb4, Ndufs4, Ndufs7, Pdhb, Slc25a11, Hadhb, Vdac3) (quantification: proteins pull-down and proteomics, *p* < 0.05; two-sample *t*-test; STRING Network *p* < 10e-16; Figures 6C,D). The third group are proteins with altered interactions with mutant ataxin-3 vs. normal ataxin-3 which belong to functional set related to transport of mitochondria (Letm1, Sfxn3, Vdac2, Slc25a12, Gdap1, Ywhaq, Ndufa13, Ywhab, Atp5mf, Atp5me, Camk2a, Slc25a4, Slc25a22, Opa1, Csnk2a2, Vps35, Atp5k, Atp5pd, Atp5f1a, Atp5pb, Atp5f1c, Hsp90aa1, Aifm1) (quantification: proteins pull-down and proteomics *p* < 0.05; two-sample *t*-test; STRING Network *p* < 10e-16; Figures 6E,F).

### 2.7. Transport of mitochondria is slowed in neurites of Ki91 and Ki150 SCA3 cerebellar neurons

**Figure 7:**
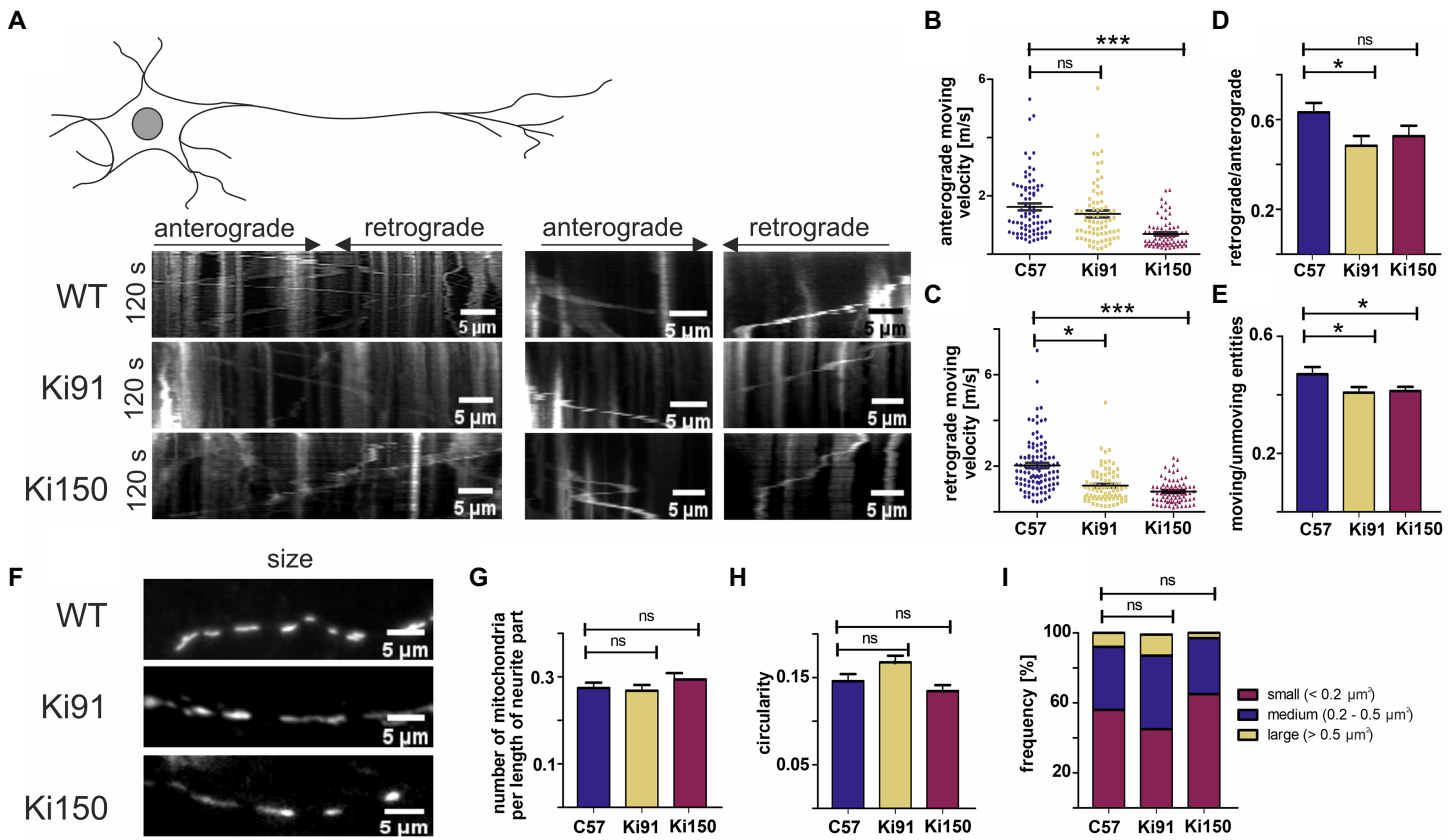
Transport of mitochondria is slower in neurites of SCA3 cerebellar neurons *in vitro*. **(A)** Representative kymographs of mitochondrial transport in primary cerebellar neurons and pictures of mitochondria size. **(B)** The average velocity (calculated as travelling distance/time) in the anterograde direction (from soma) was significantly lower in Ki150 neurites in comparison to WT. **(C)** The average velocity (calculated as travelling distance/time) in the retrograde direction (toward soma) was significantly lower in both Ki91 and Ki150 neurites in comparison to WT. **(D)** The ratio of mitochondria moving retrogradely to mitochondria moving anterogradely is lower in Ki91 neurites compared to WT. **(E)** The number of moving mitochondria is significantly lower in both Ki91 and Ki150 neurites as compared to WT. **(F)** The number of mitochondria per neurite length was not changed. **(G)** The circularity of mitochondria indicating fusion/fission events is not altered. **(H)** The average area of mitochondria is approximately the same in all tested groups. **(I)** The size distribution of mitochondria in Ki91 and Ki 150 cerebellar neurons is comparable to WT. All parameters were quantified using ImageJ. The measurements were from 3 independent experiments, with 15–25 cells analyzed each time. Two-sample *t*-test (*p < 0.05, **p < 0.01, ***p < 0.001), error bars: SEM.

Our experiments on the Ki150 and Ki21 demonstrated that using brain tissue selectively expressing human mutant or normal ataxin-3 identifies loss of binding by specific functional sets of protein to the mutant protein. We demonstrated that the presence of mutant ataxin-3 protein results in weakened or loss of interaction with CCT chaperone complexes responsible for transport along neurits, several calcium binding proteins and kinases which were strongly downregulated. The largest group of proteins with affected interaction were mitochondrial proteins and proteins involved in their transport along axons. Therefore, after identification of such a consistent set of proteins, we determined whether the loss of protein interactions in Ki150 mice results in functional impairment of mitochondrial transport in SCA3 neurites. We have selected granule neurons as a neuronal model since our previous finding demonstrated aberrant energy metabolism in these cells (Wiatr et al., 2021). Granule cell culture provides the homogenous population of neurons in culture, equal timeline, length, and way of outgrowth of neurites which prevents variability in the precise tracing of live mitochondria. We quantified the mitochondrial motility in cerebellar neurons containing only mutant ataxin-3 (Figure 7). Primary cerebellar neurons were obtained from either control (C57BL6) mice or SCA3 mice, including Ki91 knock-in with 91 CAGs in the ataxin-3 gene and Ki150 (around 150 CAG repeats). The experiment was conducted using neuronal cultures isolated from brain of three different sets of P2 pups, *N* = 3 per genotype and the neurons were cultured for 7 days prior the experiment. We identified mitochondria in neurons by specific staining with the fluorescent dye MitoTracker® Deep Red (ThermoFisher) and examined their dynamics using live imaging. For the analysis, we included only long, distinct neurites that were clearly extended from a soma and excluded neurites that were discontinued or for which we were not able to identify the soma of origin. Neurons were incubated with MitoTracker® dye (ThermoFisher) for 25 min and imaged every second over 120 s on the heated microscopic stage (37°C).

We found that trafficking of mitochondria was impaired in primary cerebellar neurons from SCA3 mice (Ki91 and Ki150) compared to control. The velocity of mitochondria was calculated by dividing the total distance by the total time of mitochondrial movement during the observation period (Figure 7).

In SCA3 neurites, we observed significantly decreased average velocity of mitochondrial movement in the retrograde direction (1.17 ± 0.09 μm s ^− 1^, mean ± SEM in Ki91 and 0.89 ± 0.06 μm s ^− 1^, mean ± SEM in Ki150) vs. the WT neurites (2.06 ± 0.13 μm s ^− 1^, mean ± SEM, *p* < 0.0001) (Figure 7C). Notably, we found that the average velocity of mitochondria in the retrograde direction decreased when the CAG repeat length increased (1.17 ± 0.09 μm s ^− 1^ − in Ki91 vs. 0.89 ± 0.06 μm s ^− 1^ in Ki150) (Figure 7C). Thus, the retrograde mitochondrial transport along neurites is slowed down in the presence of mutad ataxin-3 and the degree of diminished motility is dependent on the CAG repeat length.

A decrease in the average velocity of anterograde-moving mitochondria was also observed in Ki150 neurites (0.69 ± 0.06 μm s ^− 1^, mean ± SEM) vs the WT neurons (1.62 ± 0.12 μm s ^− 1^, mean ± SEM, *p* < 0.0001), but not in Ki91 (1.38 ± 0.12 μm s ^− 1^, mean ± SEM, *p* = 0.16) (Figure 7B). Consistent with those results, the ratio of the mitochondria moving retrogradely vs. the mitochondria moving anterogradely was significantly decreased only in Ki91 neurites (0.5 ± 0.05, mean ± SEM) as compered to WT neurites (0.66 ± 0.04, mean ± SEM, *p* < 0.015, Figure 7D). Mitochondria not only moved slower in SCA3 neurites, they also were slightly more static as demonstrated by ratio of moving vs. unmoving entities (0.41 ± 0.02, mean ± SEM in Ki91 and 0.41 ± 0.02 μm s ^− 1^, mean ± SEM in Ki150 compared to 0.47 ± 0.02, mean ± SEM in WT *p* < 0.05, Figure 7E).

The impairment of mitochondrial trafficking in SCA3 neurites was not accompanied by any significant changes regarding the number, shape, or size of the mitochondria in primary cerebellar SCA3 neurons (Figures 7F–I). We did not detect any differences in the number of mitochondria normalized to the length of the measured neurite (*t*-test, *p* < 0.05; Figure 7G). There was also no change in the mitochondrial shape, which is related to fusion/fission events and which was calculated by dividing the width and height of each mitochondrion (*t*-test, *p* < 0.05; Figure 7H). Distribution of the mitochondria size was similar across all tested groups (*t*-test, *p* < 0.05; Figure 7I).

## 3. Discussion

The proteins containing long polyQ tracts are often pleiotropic and may either acquire new or lose the functions of the original protein. Ataxin-3 and its mutant counterpart is a particularly explicit example of a protease and deubiquitinase involved in nearly all cellular processes associated with protein degradation. Such pleiotropy is reflected by broad influence of mutant ataxin-3 on proteome and phosphoproteome (Wiatr et al., 2019, 2021). Therefore, it is very difficult to pinpoint a network of key pathogenic mechanism and to find the cure. Moreover the mouse models offered in the polyQ research are always compromised in their features such as lack of robust combination of preclinical, motor, molecular and neuropathological properties. For instance, the mild or moderate motor and molecular phenotype, accuracy of genetic constructs, full length human allele, and the concomitant presence of mouse allele are always compromised (Cemal et al., 2002; Switonski et al., 2015; Haas et al., 2022). The ideal model would have a quick phenotype for preclinical tests, have an entire human protein, uninterrupted CAG tract in mRNA and expansion phenotype. We previously generated Ki91 SCA3 mouse model (Wiatr et al., 2019, 2021), and slightly increased the CAG repeat count (110–120) resulting in the model that has demonstrated many prominent SCA3 alterations. However, for conducting swift preclinical testing, an additional and more severe SCA3 model with aggravated phenotype is in demand. Therefore using the Ki91 model, its expansion feature and the selective breeding, we obtained the Ki150 SCA3 model displaying a long CAG (150–170) tract in the humanized *ATXN3* gene and more severe phenotype. The Ki150 mice present deteriorated motor phenotype already at the age of 4 weeks and formation of early ataxin-3 inclusions in various brain tissues, which are large and dense. The neuropathology in Ki150 and the solid inclusions are compatible to investigate the mutant ataxin-3 pleiotropy, and the extensive presence of various protein complexes and aggregates. Considering that the binding of mutant ataxin-3 with long polyQ tract to different proteins may be intensified we aimed at identifying protein–protein interactions of normal and mutant ataxin-3. This would provide valuable information on proteome changes in the brain and the resulting pathogenic protein network of SCA3 which could later be connected to affected neuronal function. The protein partners of ataxin-3 identified so far came from several studies performed mostly in artificial overexpression system *in vitro* or non-mammalian animal models (Mazzucchelli et al., 2009; Sowa et al., 2009; Kristensen et al., 2018; Weishäupl et al., 2019; Antonicka et al., 2020). We determined ataxin-3 protein interactions in an *in vivo* pathogenic system consisting of Ki150/150 (mutant human ataxin-3) and Ki21/21 (normal human ataxin-3).

In order to find proteome differences in the Ki21 vs. Ki150 models, we performed orthogonal IEC/SEC fractionation of Ki21 and Ki150 brain lysates. Protein complexes associated with mutant ataxin-3 were generally identified in heavier fractions compared to ataxin-3 with normal polyglutamine tract suggesting an intensive impact of mutant protein on the increased formation of protein–protein complexes. We subjected the samples to mass-spec analysis to identify the protein composition of fractions enriched in ataxin-3. We discovered highly deregulated proteins in protein fractions belonging to molecular chaperone CCT/TRiC complex which is the key regulator of protein aggregation of polyQ proteins (Darrow et al., 2015; Grantham, 2020). Downregulation of the TCP1 complex is linked to be common in neurodegenerative disorders such as Alzheimer’s, and Huntington disease. The autophagic flux is reduced by compromise of individual CCT subunits, effect of loss-of-CCT activity on aggregation is primarily a consequence of autophagy inhibition (Pavel et al., 2016). Moreover, our results suggest that ataxin-3 shows a tendency to interact with subunits of protein complexes of a circular structure, such as CCT, proteasome and Camk2 subunits (Supplementary Figure 3). A disrupted interaction of mutant ataxin-3 with subunits of complexes of circular structure might lead to increase in formation of self ataxin-3 annular aggregates, which enables the transition to more toxic fibrillar aggregates and their precipitation in the form of inclusions. It must be noted that, e.g., Camk2a *in vivo* forms homo-12-mers of >600 kDa. This suggests that the extension of the polyQ tract in ataxin-3 affects protein oligomerization resulting from either incomplete folding or degradation. The same applies to the aforementioned chaperones and proteasome, whose fully-functional oligomers cannot appear in the 282–218 kDa fractions.

As a complementary and more direct approach, we aimed to identify any loss or gain of protein interaction of mutant ataxin-3 (150 CAG repeats) by performing co-immunoprecipitation in two representative brain regions: the cerebellum and cerebellar cortex which we previously demonstrated to be relevant for SCA3 (Wiatr et al., 2019, 2021). We found that many protein interactions are lost due to the presence of extended polyQ tract in the ataxin-3. The top result identified by two different strategies both in cerebellum and cerebral cortex was Calcium/Calmodulin Dependent Protein Kinase II alpha and beta (Camk2a and b), which plays a pivotal role in mitochondrial transport in axons (Woolums et al., 2020). Moreover, we identified impaired interactions of proteins involved in mitochondrial function, mitochondrial transport, translation, proteasome, and cytoskeleton.

We focused on the functional consequences of the altered protein interactions with ataxin-3 for the mitochondrial transport in neurons. The impairment of mitochondrial transport would correspond well with our previous findings, including impaired energy metabolism and mitochondrial parameters in SCA3 mouse primary cerebellar neurons, a number of dysregulated mitochondrial proteins in SCA3 cerebellum, and mislocalization of mitochondrial proteins in SCA3 axon (Wiatr et al., 2021). Moreover, an enhanced interaction of several mitochondrial proteins with mutant ataxin-3 have been demonstrated in an overexpression cellular model (Kristensen et al., 2018).

To clarify whether motility of mitochondria is disturbed in SCA3 cerebellar neurites, we conducted a quantitative analysis of the mitochondrial transport in cultured primary cerebellar neurons established from Ki91 and Ki150 knock-in mouse models. For the first time, we demonstrate in a direct manner that the transport of mitochondria is disturbed in SCA3 cerebellar neurites. Due to the complex and elongated structure of a neuron, perpetual delivery of cellular components between cell body and neuronal projections pose a unique challenge. Mitochondria are one of the crucial organelles for neuronal function that need to be replenish efficiently, since they are exposed to damaging influence of reactive oxygen species and age over time (Nicholls, 2004; Balaban et al., 2005; Tao et al., 2014). Mitochondria are produced primarily in the cell body and are delivered *via* microtubule-based transport to the neuronal projections (Davis and Clayton, 1996; Schwarz, 2013; Misgeld and Schwarz, 2017). Impairment of axonal mitochondrial transport ultimately results in the insufficient local energy demand and axon degeneration (Sheng and Cai, 2012). Indeed, defects in mitochondrial transport are associated with a number of neurodegenerative diseases, including Alzheimer’s, Huntington’s, and Parkinson’s disease, ALS, Charcot–Marie-Tooth (Piccioni et al., 2002; Sheng and Cai, 2012, p. 2; Shirendeb et al., 2012; Itoh et al., 2013; Kalmar et al., 2017; Stykel et al., 2018; Otomo et al., 2022). Moreover, mitochondrial impairment likely plays a role in SCA3 pathogenesis (Da Silva et al., 2019).

Here, we hypothesize that the loss of interaction of ataxin-3 with two protein complexes of a circular structure, namely CaMKII and CCT (chaperonin containing TCP-1)/TCP-1/TRiC is disturbing mitochondrial transport in SCA3 neurites. CaMKII is a key regulator of calcium signaling which is pivotal in the regulation of axonal transport of mitochondria (Breuer and Atkinson, 1988; Saotome et al., 2008; Wang and Schwarz, 2009, p. 2; Brault et al., 2019). CaMKII controls mitochondrial translocation in smooth muscle and imbalance of CaMKII-dependent Ca^2+^ entry lead to disruption of mitochondrial axon transport and ultimately axonal degeneration (Woolums et al., 2020, p. 4) (Nguyen et al., 2018). In line with our hypothesis, the activity of the CaMKII is likely affected by the presence of mutant ataxin-3, as we have previously shown decreased phosphorylation of its many protein target sites and the CaMKII protein level dysregulation in SCA3 mouse brain (Wiatr et al., 2019). Of note, intracellular Ca^2+^ levels are also modulated by the presence of mutant ataxin-3 (Pellistri et al., 2013).

In addition, CCT (chaperonin containing TCP-1)/TCP-1/TRiC subunits enhance retrograde axonal transport by modulating tau phosphorylation of the AT8 phospho-epitope (Ser199, Ser202, Thr205) (Zhao et al., 2016). The AT8 epitope is crucial in regulating mitochondrial movement as its replacement with phosphomimetic aspartates lead to inhibition of mitochondria transport in the neurites of PC12 cells (Shahpasand et al., 2012). Tau hyperphosphorylation was also shown to be linked with reduced number of mitochondria in cortical axons (Rodríguez-Martín et al., 2016). Therefore, dysregulation of CCT/TCP-1/TRiC complex subunits resulting from compromised interaction with mutant ataxin-3 might have a direct impact on mitochondrial transport in axons. In addition to disrupted interaction of mutant ataxin-3 with CaMKII and CCT/TCP-1/TRiC, the presence of ataxin-3 inclusions which we previously shown in cereberal axons could also perturb mechanisms of axonal transport (Wiatr et al., 2021, p. 21).

In conclusion, to understand the SCA3 neuropathomechanism in our model we investigated the functional consequences of the expansion of CAG tract to over 150 repeats by discovering ataxin-3 mutant protein interactions and their influence on cellular structures. Guided by specific sets of identified protein interactors we investigated live mitochondrial motility in Ki150 neurites. Based on our study we demonstrate a preclinical grade SCA3 knock-in model with intense neuropathology, leading to altered interactions and live organelle transport impairment in neurites.

## 4. Materials and methods

### 4.1. Animals

Maintaining and breeding were performed at standard conditions with an 18/6-h light/dark cycle and water and food *ad libitum*. The animals were marked using numerical ear tags (National Band & Tag Company, Newport, USA). The stress level of the animals was minimized throughout all procedures and animal handling. The animal experimentation and handling were approved and monitored by the Local Ethical Commission for Animal Experiments in Poznan. The animals were sacrificed according to AVMA Guidelines for the Euthanasia of Animals by placing them in the programmable CO2 chamber (Rothacher Medical, Heitenried, Switzerland). SCA3 Ki91 and Ki150 mice (C57BL/6 background) and age-matched C57 and Ki21 controls were used for co-immunoprecipitations. The cerebellum and cerebral cortex for proteomic analysis were collected from 2 month-old animals.

### 4.2. Western blot analysis

Samples were harvested from mouse brain regions and homogenized in the buffer containing 60 mM TRIS-base, 2% SDS, 10% sucrose, and 2 mM PMSF, followed by bath sonication. The protein concentration was estimated using a Pierce BCA protein assay kit (Thermo Scientific, Rockford, IL, USA). For each analysis, 25 μg of total protein was diluted in a loading buffer containing 2-mercaptoethanol and boiled for 5 min. The proteins were separated by SDS–polyacrylamide gel electrophoresis (5% stacking/ 10% resolving gel), transferred to nitrocellulose, and stained with Ponceau S solution. The blots were blocked with 5% non-fat milk in PBS/0.05% Tween 20 for 1 h at RT and, subsequently, incubated for 24 h at 4°C with the following primary antibodies: anti-ataxin-3 (1:2000; ProteinTech, Rosemont, IL, USA) and LaminB (1:2000; ProteinTech, Rosemont, IL, USA). The blots were probed with the respective HRP-conjugated secondary antibody (anti-rabbit or anti-mouse, 1:2000; Jackson ImmunoResearch, Suffolk, UK). The immunoreaction was detected using the ECL substrate (ThermoFisher Scientific, Waltham, MA, USA).

### 4.3. Immunofluorescence staining

The animals were deeply anesthetized and transcardially perfused using saline, followed by 4% PFA. The brains were removed, post-fixed in 4% PFA for 48 h, and cryopreserved with graded sucrose (10–20–30%) over 72 h. The 20-μm parasagittal mouse brain sections were cut using a cryostat at −20°C and collected on SuperFrost Plus slides (Thermo Fisher Scientific, Waltham, MA, United States). The sections were processed immediately. The HIER procedure was applied by incubation of the sections in citrate buffer (pH 9.0) for 30 min at 60°C. The sections were blocked *via* incubation in 4% normal goat serum in TBS for 1 h. For immunofluorescent staining, the sections were incubated overnight at 4°C, rabbit anti-ataxin-3 (1:200; ProteinTech, Rosemont, IL, United States), and subsequently with the anti-rabbit antibody labeled by AlexaFluor488, or AlexaFluor647 (1:400; Jackson ImmunoResearch; Suffolk, United Kingdom). The sections were end-stained with Hoechst 33342 (Sigma) nuclear stain at 1:1,000 and embedded in Fluoroshield (Sigma) mounting medium. For aggregates quantification Aggrecount v1.3 software was used and the applied parameters are available in Supplementary File 1. As an input file, we used 3 individual images of the same size per biological replicate *n* = 4. In the setup file, the parameter “Mean” (Mean fluorescent intensity from a cell ROI) was used for aggregate intensity and “aggregates” value denoted a total number of aggregates.

### 4.4. Co-immunoprecipitation

Sacrificed animals were decapitated, afterward, the cerebral cortex and cerebellum were dissected from the brain and snap freeze in liquid nitrogen and then stored at-80 ° C. A direct immunoprecipitation approach was performed with the primary mouse anti-ataxin-3 antibody 1H9, or negative control IgG (AC011; Santa Cruz Biotechnology, sc2025) and Dynabeads™ Co-Immunoprecipitation Kit (14321D, Thermo Fisher Scientific). To antibody coupling used 6 μg/mg beads, antibody coupling was performed according to the manufacturer’s protocol. Briefly, Dynabeads were washed in C1 buffer then incubated overnight in C1, C2, and antibodies at 37 ° C. On the next day, antibodies coupled to the beads were washed in HB, LB, and SB buffers. After washing beads were stored until use in the SB buffer in the fridge. The lysis buffer from the kit was modified with 100 mM NaCl, 2 mM MgCl2, 1 mM DTT, and 40 μM PMSF. The rest procedure was performed according to the manufacturer’s instructions, beads were incubated with the lysates for 30 min. at 4°C, to elute the protein complexes HPH EB (0.5 M NH4OH, 0.5 mM EDTA) was used. After elution samples were lyophilized in a speed vac overnight. Then the samples were directly digested for the mass spectrometry.

### 4.5. Protein purification

Whole brains from Ki21 and Ki150 mice were collected and snap froze in liquid nitrogen. Afterward, brains were homogenized using stainless steel beads in Allshend Bioprep-6 homogenizer in PBS EDTA 5 mM buffer supplemented with Halt™ Protease Inhibitor Cocktail (Thermo Fisher Scientific). Samples were homogenized 2× 30s 3,5 k RPM, between homogenization rounds samples, were placed on ice. After homogenization samples were centrifuged to collect supernatants. The clear supernatants were applied onto an ion-change CM-cellulose resin (negatively charged), next the flowthrough was applied onto DEAE-cellulose resin (positively charged) and the flowthrough was collected. The collected flow-through was concentrated to ∼2.5 ml and applied on size exclusion HiLoad Superdex 200 16/60 column (GE Healthcare) connected to the AKTA FPLC system (Amersham Biosciences). The size exclusion chromatography was run as the final step of purification in a buffer composed of PBS EDTA 50 uM, to yield 2 ml in each protein fraction.

### 4.6. Vacuum dot immunoblot

The nitrocellulose membrane and Whatman filters (all GE Healthcare, Chicago, Il, USA) were washed 2X with Milli-Q H20 and 2X with Tris-Buffered Saline (TBS) by vacuum filtration. 450uL of each fraction sample was spotted on nitrocellulose membrane and washed 2X with TBS by vacuum filtration, and then allowed to air-dry. The blots were stained with Ponceau S solution and blocked with 5% nonfat milk in PBS/0.05% Tween 20 for 1 h at RT and subsequently incubated at 4°C overnight with the following primary antibodies: anti-ataxin-3 (1:2000; ProteinTech, Rosemont, IL, USA) and LaminB (1:2000; ProteinTech, Rosemont, IL, USA). The blots were probed with the respective HRP-conjugated secondary antibody (anti-rabbit or anti-mouse, 1:2000; Jackson Immuno Research, Suffolk, UK). The immunoreaction was detected using the ECL substrate (Thermo Fisher Scientific, Waltham, MA, USA). Data was collected using ChemiDoc XRS+ System with Image Lab v5.2 Software (Bio-Rad). To avoid overexposure of any band or dots, image acquisition times were set based on image histograms. Images were not processed before quantitation. Data within a membrane were normalized to total protein (Ki21 vs. Ki150).

### 4.7. Mass spectrometry

The lyophilized samples were dissolved in 100 mM sodium bicarbonate, and then 5ug protein from each sample was taken to further processing. Volume containing 5ug protein was reduced in DTT for 5 min at 95°, then placed on ice, and then acylated in IAA for 20 min in dark at room temperature, afterward samples were digested by trypsin overnight at 37°C in a water bath. The analysis of the proteome was performed with the use of the Dionex UltiMate 3,000 RSLC nanoLC system connected to the Q Exactive Orbitrap mass spectrometer (Thermo Fisher Scientific, Waltham, MA, USA). Peptides derived from in-solution digestion were separated on a reverse-phase Acclaim PepMap RSLC nanoViper C18 column (75 μm × 25 cm, 2 μm granulation) using acetonitrile gradient (from 4 to 60%, in 0.1% formic acid) at 30°C and a flow rate of 300 nL/min (for 230 min). The spectrometer was operated in data-dependent MS/MS mode with survey scans acquired at a resolution of 70,000 at m/z 200 in MS mode, and 17,500 at m/z 200 in MS2 mode. Spectra were recorded in the scanning range of 300–2000 m/z in the positive ion mode. Higher energy collisional dissociation (HCD) ion fragmentation was performed with normalized collision energies set to 25. Swiss-Prot mouse database was used for protein identification with a precision tolerance set to 10 ppm for peptide masses and 0.08 Da for fragment ion masses. Raw data obtained for each dataset were processed by MaxQuant 1.5.3.30 version for protein identification and quantification. Protein was considered as successfully identified if the Andromeda search engine found at least two peptides per protein, and a peptide score reached the significance threshold FDR = 0.01.

### 4.8. Live imaging of mitochondrial transport

Primary neurons were obtained from P2 pups and cultured as previously described (Wiatr et al., 2021). Briefly, primary neuron cultures were derived from Ki150, Ki91 and C57 mice according to AVMA Guidelines for the Euthanasia of Animals. Followed by trypsin digestion, cells were washed in HBSS (Thermo Fisher Scientific, Waltham, MA, United States) with 1× penicillin–streptomycin (Thermo Fisher Scientific, Waltham, MA, United States), and then in a plating medium (Dulbecco’s modified Eagle medium, 1× CTS GlutaMAX-I supplement, 1× penicillin–streptomycin (all Thermo Fisher Scientific, Waltham, MA, United States)). Clumps of tissue debris were allowed to settle down in a tube, and the supernatant containing dissociated cells was centrifuged for 3 min at 1,300 rpm. Cells were seeded onto poly-D-lysine (Thermo Fisher Scientific, Waltham, MA, United States) covered plates. Neurons were maintained in conditioned Neurobasal medium (Thermo Fisher Scientific, Waltham, MA, United States) supplemented with 2% B27 supplement, 1× CTS GlutaMAX-I supplement, 1× penicillin–streptomycin (all Thermo Fisher Scientific, Waltham, MA, United States), 1× apo-transferrin (Sigma-Aldrich, St. Louis, MO, United States), and N2 (0.005 mg/ml insulin, 0.0161 mg/ml putrescin, 30 nM Na-Selenite, 51 nM T3, 20 nM progesterone) in a humidified incubator with 5% O2 and 5% CO2 in air at 37°C. The maintenance medium was replenished with half-feed changes every 2–3 days.

To track mitochondria movement cerebellar neurons at DIV7 were incubated with 30 nM of MitoTracker® Deep Red (ThermoFisher) at 37°C for 25 min before imaging. Cells were then quickly rinsed with PBS and imaged to track mitochondrial trafficking. The series of time-lapsed images were captured at 1.0 frame/2 s for 2 min. The images were taken under a 63X objective using Opera LX (PerkinElmer) spinning disc microscope with a heated (37°C), 5% CO2-controlled stage. To analyze and quantify the movement of the mitochondria kymographs were generated with the open-source image-processing package Fiji/ImageJ. First, the “rectangular selection” function was used to choose the neurite region to be analyzed. Next, the “reslice” function was used to compress each cropped image to one line. Then, the “z-projection” function was used to compile all time-lapse image converted lines into one single picture. In each kymograph, the x-axis represents mitochondrial displacement, and the y-axis represents time. Vertical lines indicate stationary mitochondria while diagonal lines are moving mitochondria (Course et al., 2017). To asses if there were any alterations in mitochondria morphology, the area, width and height of individual organelles were measured. Lastly, the number of mitochondria normalized to the length of the measured neurite was calculated. The analysis was performed in 3 independent experiments, N = 3 per genotype. All statistical analyses were performed using the Student’s *t*-test and are presented as mean ± SEM.

### 4.9. Statistical analysis

The data obtained in behavioral tests were subjected to a two-way ANOVA, followed by Bonferroni post-tests. value of *p*s of less than 0.05 were considered significant. Identification of proteins on raw proteomic data was performed by the Andromeda search engine in Mascot using the following inclusion criteria: at least two different peptides per protein were identified per sample, and a total peptide score reached the significance threshold FDR = 0.01. Identified proteins matching the inclusion criteria were subjected to further statistical analysis with a two-sample *t*-test, and dysregulation of protein level reaching value of *p* < 0.05 was considered as significant.

## Author’s note

Imaging was performed using the facilities of the High-throughput Screening Laboratory (IBCh, PAS, Poland). Proteomic mass spectrometry analyses were performed in the Laboratory of Mass Spectrometry and the library synthesis was performed in the Laboratory of Genomics (European Centre for Bioinformatics and Genomics; ECBiG; IBCH, PAS, Poznań, Poland). Primary neuron cultures were maintained in the Cell and Tissue Culture Laboratory (IBCh, PAS, Poland).

## Data availability statement

The original contributions presented in the study are included in the article/Supplementary material, further inquiries can be directed to the corresponding author.

## Ethics statement

The animal study was reviewed and approved by Local Ethical Commission for Animal Experiments in Poznań.

## Author contributions

PP and KW performed live animal behavioral experiments. KW performed live-imaging experiments in neurons and data analysis. PP and ŁM performed all proteomic experiments and analysis of proteomic data. PP and MR performed chromatography experiments. YT provided anti-Ataxin-3 1H9 antibody and revised the manuscript. MF, PP, KW, and MR wrote the manuscript. MF conceived, supervised all experiments, analyzed the data and was responsible for research concept and obtaining funding. All authors contributed to the article and approved the submitted version.

## Funding

This work was supported by European Research Projects on Rare Diseases (JTC 2017), National Center for Research and Development, Poland [Grant Numbers: ERA-NET-E-RARE-3/III/TreatPolyQ/08/2018 (to MF and YT)], National Science Centre, Poland (OPUS grant: 2021/41/B/NZ2/03881 to MF; ETIUDA grant: 2019/32/T/NZ3/00504 to KW), and a Grant of National Ataxia Foundation: Pioneer SCA3/MJD Translational Research Award #688790 (to MF and YT).

## Conflict of interest

The authors declare that the research was conducted in the absence of any commercial or financial relationships that could be construed as a potential conflict of interest.

## Publisher’s note

All claims expressed in this article are solely those of the authors and do not necessarily represent those of their affiliated organizations, or those of the publisher, the editors and the reviewers. Any product that may be evaluated in this article, or claim that may be made by its manufacturer, is not guaranteed or endorsed by the publisher.

## Abbreviations

ANOVA: Analysis of variance
CCT: Chaperon Containing TCP1-complex
DIV: Days *in vitro*
MJD: Machado-Joseph disease
MS: Mass spectrometry
mo: Month-old
polyQ: Polyglutamine
SCA3: Spinocerebellar ataxia type 3
SEM, Standard error of mean
IEC: Ion exchange chromatography
SEC: Size using size exclusion chromatography
WT: Wildtype
