# Supplementary material for: Impaired interactions of ataxin-3 with protein complexes reveals their specific structure and functions in SCA3 Ki150 model
Source: Front Mol Neurosci. 2023 Mar 24;16:1122308. doi: 10.3389/fnmol.2023.1122308 (PMC10080164; doi:10.3389/fnmol.2023.1122308)
Supplement: Supplementary file 6 [file Data_Sheet_1.docx]

**Supplementary figure 1. Exemplified sagittal full section of Ki150 brain for overview of brain structures containing ataxin-3-positive aggregates.**

(A) Exemplified entire section of Ki150 mouse brain fluorescently labeled with anti-ataxin-3 antibodies in high resolution. The section demonstrates the regional localization of inclusions occurring in high numbers across the whole brain slice of 8-month-old Ki150. The image was acquired on high throughput microscope. One section of the composite image is the equivalent of 640 um (the entire section comprises approximately 12 mm). (B) Higher magnification images (scale bars) of inclusion bodies in cerebellum, cerebral cortex, striatum, hippocampus, pons, and olfactory bulb.

**Supplementary figure 2. Chromatography of the Ki21 and Ki150 mouse brain lysates.**

(A) Western blot after ion exchange chromatography DEAE-cellulose (positively charged resin) and CM-cellulose (negatively charged resin). The majority of the mutant ataxin-3 complexes in Ki150 brain lysate is present in the flow-through from both resins. In addition, the figure demonstrates that after collecting the flow through containing ataxin-3 and after subsequent application of a relatively high-salt buffer to the same column (to enforce the release of proteins), collection of any more ataxin-3 protein does not occur - ataxin-3 can hardly be detected in 50 mM NaCl buffer and can not identified in 100 and 150 mM NaCl buffer.

(B) SEC Elution profile (A280 in mAU). The SEC analysis was performed by injecting 2.2 mL sample volume onto a Superdex 200 16/60 column connected to AKTA chromatography system. Total number of biological replicates: n = 6, n = 3 per genotype

**Supplementary figure 3. Mutant ataxin-3-interacting complexes and their common type of circular quaternary structure.**

Protein complexes that interact with mutant ataxin-3 show similar circular character of their crystal structure. The graphics demonstrates circular complexes of proteasome, Camk2a, Camk2b, TRiC/CCT subunit 5, and circular complexes within mitochondrial ATPase. All complexes are depicted using the same scale.
