# Supplementary figures and images for: Impaired interactions of ataxin-3 with protein complexes reveals their specific structure and functions in SCA3 Ki150 model
Source: Front Mol Neurosci. 2023 Mar 24;16:1122308. doi: 10.3389/fnmol.2023.1122308 (PMC10080164; doi:10.3389/fnmol.2023.1122308)

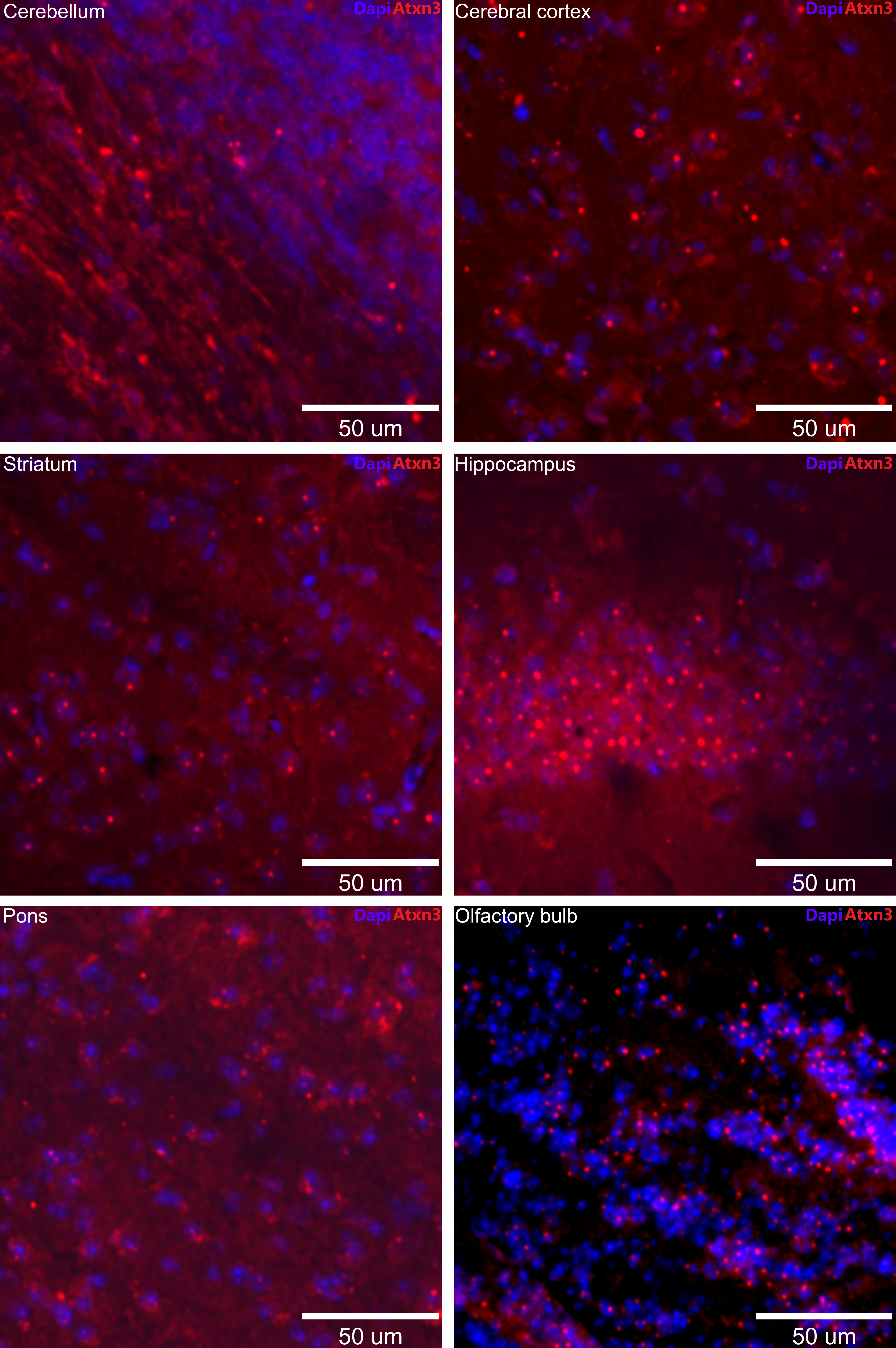

Supplement: Supplementary file 8 [file Image_2.JPEG]

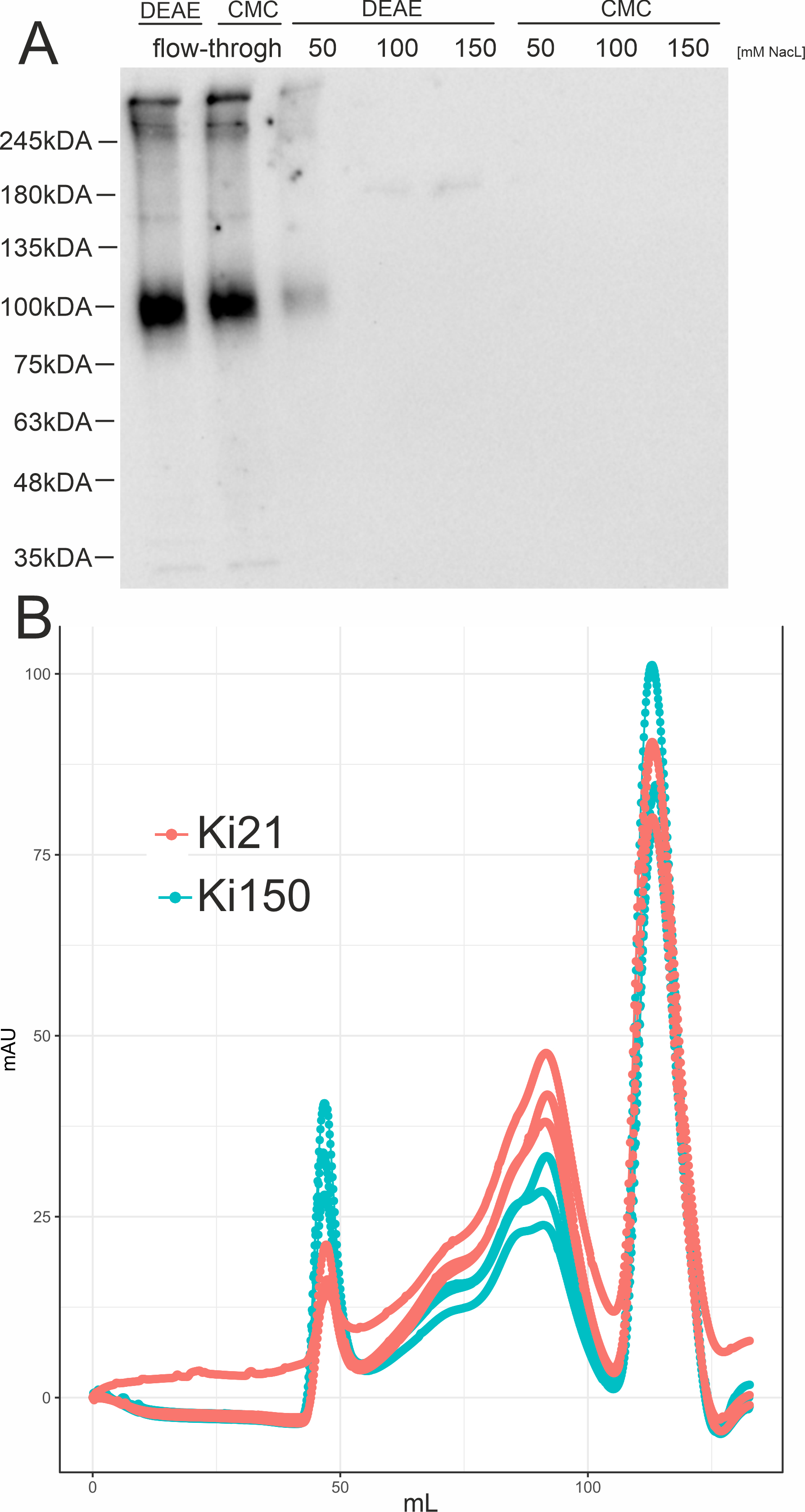

Supplement: Supplementary file 9 [file Image_3.JPEG]

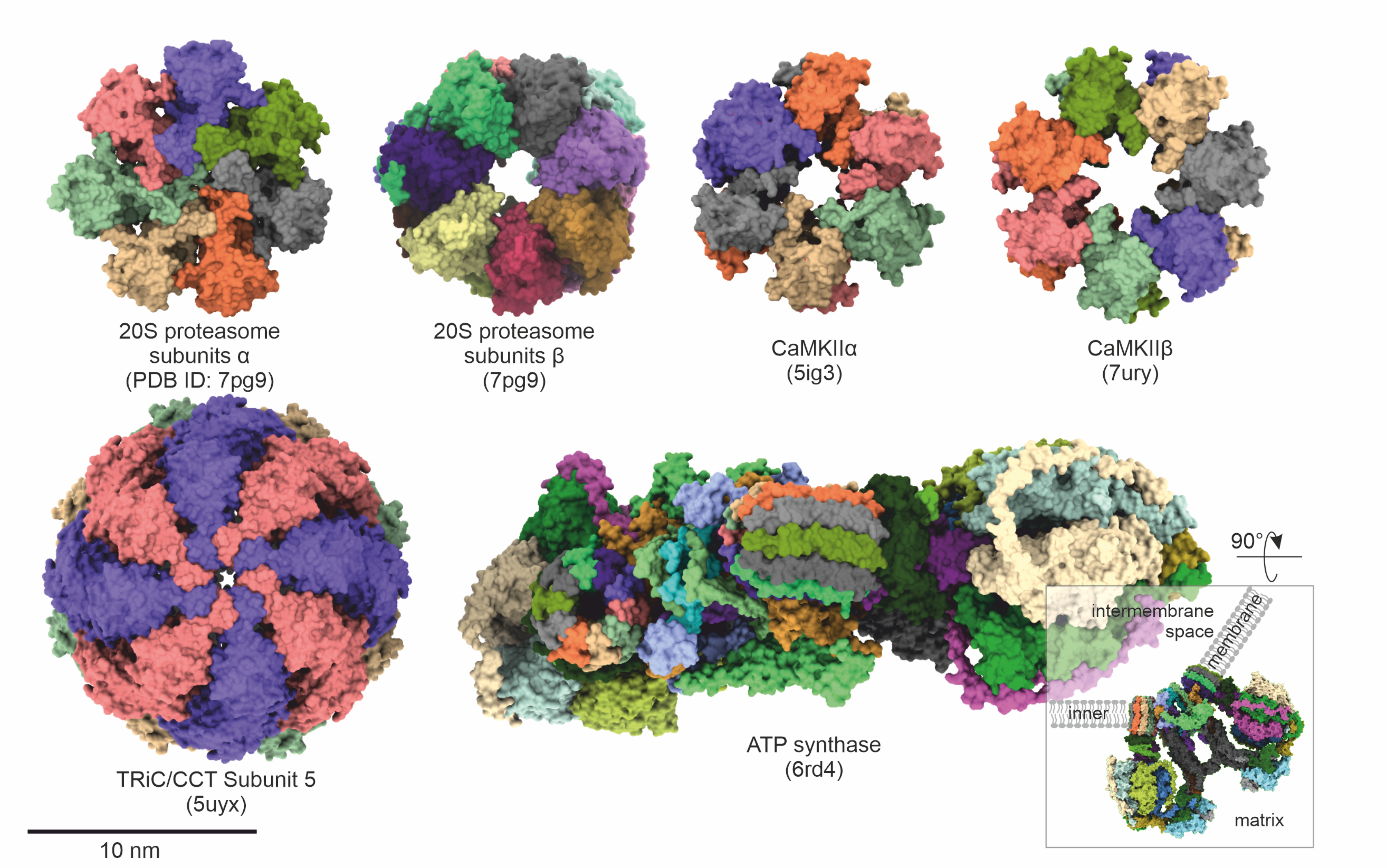

Supplement: Supplementary file 10 [file Image_4.JPEG]
